# Supplementary material for: The Systems Biology Research Tool: evolvable open-source software
Source: BMC Syst Biol. 2008 Jun 29;2:55. doi: 10.1186/1752-0509-2-55 (PMC2446383; doi:10.1186/1752-0509-2-55)
Supplement: Additional file 1 — SBRT Archive. An archive of the current version of the Systems Biology Research Tool. [file 1752-0509-2-55-S1.zip › sbrt-1.4.0/doc/developers_guide/api/sbrt/kernel/fba/proc/package-summary.html]

sbrt.kernel.fba.proc


|  |  |  |
| --- | --- | --- |
| **Overview** \| **Package** \| Class \| **Use** \| **Tree** \| **Deprecated** \| **Index** \| **Help** | |  |
| **PREV PACKAGE**   **NEXT PACKAGE** | **FRAMES**    **NO FRAMES**     **All Classes** |  |


---

## Package sbrt.kernel.fba.proc

Provides classes and interfaces for executing Flux Balance Analysis processes.

**See:**
  
          **Description**

| **Class Summary** | |
| --- | --- |
| **CatalystDeletionProcess** | This class is used to perform catalyst deletion experiments. |
| **CdHarFluxVectorProcess** | This class is used to perform coordinate direction hit-and-run flux vector experiments. |
| **CvCatalystDeletionProcess** | This class is used to perform constraint-variation catalyst deletion experiments. |
| **CvFbaOptimizationExperiment** | This class is used to perform constraint-variation FBA optimization experiments. |
| **CvFbaOptProcess<R>** | This class is used to represent constraint-variation Flux Balance Analysis experiments. |
| **CvFluxIntervalProcess** | This class is used to perform constraint variation flux interval experiments. |
| **CvObjectiveFunctionProcess** | This class is used to perform constraint-variation objective function experiments. |
| **CvRxnDeletionProcess** | This class is used to perform constraint-variation reaction deletion experiments. |
| **CycleIdProcess** | This class is used to execute steady state cycle identification processes. |
| **ExtremeCurrentProcess** | This class is used to perform extreme current experiments. |
| **FbaOptimizationProcess** | This class is used to perform basic Flux Balance Analysis optimization. |
| **FbaProcess<R>** | This abstract class is used to represent Flux Balance Analysis experiments. |
| **FbaSolverProcess** | This class is used to solve Sv = 0. |
| **FluxActivityProcess** | This class is used to perform flux activity experiments. |
| **FluxActivityProcess.Results** | This class is used to represent the results of a flux activity experiment. |
| **FluxCapProcess** | This class is used to perform a flux cap identification. |
| **FluxIntervalProcess** | This class is used to perform flux interval experiments. |
| **FluxomeReducerProcess<T extends Fluxome>** | This class is used to execute fluxome reduction processes. |
| **FluxPlasticityProcess** | This class is used to perform flux plasticity experiments. |
| **FluxPlasticityProcess.Results** | This class is used to represent the results of a flux plasticity experiment. |
| **InitialPointGenerator** | This class is used to perform initial point experiments. |
| **NetworkInfoProcess** | This class is used to gather basic information about a fluxome. |
| **NetworkInfoProcess.Results** | This class is used to represent the information gathered about a fluxome. |
| **ObjectiveFunctionAnalysis** | This class is used to perform objective function experiments. |
| **RandomConstraintsGeneratorV1** | This class is used to generate random flux constraints. |
| **RandomEnvGeneratorV1** | This class is used to generate random environments for use in flux balance analysis. |
| **RandomObjectiveGenerator** | This class is used to generate random objective functions. |
| **RxnDeletionProcess** | This class is used to perform reaction deletion experiments. |
| **StoichVariationProcess** | This class is used to perform stoichiometry-variation FBA optimizations. |
| **SvCdHarFluxVectorProcess** | This class is used to perform space variation coordinate direction hit-and-run flux vector experiments. |
| **SvInitialPointProcess** | This class is used to perform constraint variation initial point experiments. |

## Package sbrt.kernel.fba.proc Description

Provides classes and interfaces for executing Flux Balance Analysis processes.

---


|  |  |  |
| --- | --- | --- |
| **Overview** \| **Package** \| Class \| **Use** \| **Tree** \| **Deprecated** \| **Index** \| **Help** | |  |
| **PREV PACKAGE**   **NEXT PACKAGE** | **FRAMES**    **NO FRAMES**     **All Classes** |  |


---
